# Engineering CAR T cells for enhanced efficacy and safety

**DOI:** 10.1063/5.0073746

**Published:** 2022-01-18

**Authors:** Yiqian Wu, Ziliang Huang, Reed Harrison, Longwei Liu, Linshan Zhu, Yinglin Situ, Yingxiao Wang

**Affiliations:** 1 Institute of Engineering in Medicine, University of California, San Diego, La Jolla, California 92093, USA; 2 Department of Bioengineering, University of California, San Diego, La Jolla, California 92093, USA

## Abstract

Despite its success in treating hematologic malignancies, chimeric antigen receptor (CAR) T cell therapy faces two major challenges which hinder its broader applications: the limited effectiveness against solid tumors and the nonspecific toxicities. To address these concerns, researchers have used synthetic biology approaches to develop optimization strategies. In this review, we discuss recent improvements on the CAR and other non-CAR molecules aimed to enhance CAR T cell efficacy and safety. We also highlight the development of different types of inducible CAR T cells that can be controlled by environmental cues and/or external stimuli. These advancements are bringing CAR T therapy one step closer to safer and wider applications, especially for solid tumors.

## INTRODUCTION

Chimeric antigen receptor (CAR) T cell therapy has advanced as one of the most promising cancer treatments during the past decade especially for blood tumors.^1–5^ CAR T therapy involves *ex vivo* genetic engineering of the patients' T cells with the CAR molecule, which equips the T cells with redirected specificity against target tumor cells, and the subsequent infusion of the CAR T cells back into the patients for cancer treatment. As of September 2021, five CAR T products have been approved by the Food and Drug Administration (FDA) in the United States, targeting leukemia, lymphoma, and multiple myeloma.

**FIG. 1.**
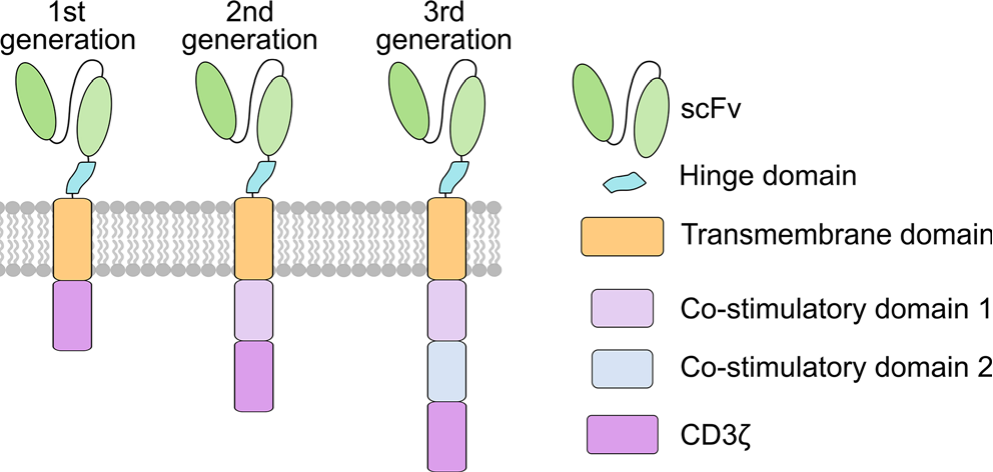
Evolution of different generations of CARs. Structures of the first, second, and third generations of CARs.

In fact, synthetic chimeric molecules composed of antibody-like variable regions fused to T cell receptor (TCR)-derived constant regions were first reported approximately three decades ago.^6–9^ These were later referred to as the first generation CAR, typically containing an extracellular single-chain variable fragment (scFv) for antigen recognition, hinge (H), and transmembrane (TM) domains for signal transduction, and an intracellular CD3z for activation (Fig. 1). Despite the antigen-specific activation and cytotoxicity, the first generation CAR T cells showed low proliferation *in vivo*. This led to the development of the second generation CAR,^10–12^ where a co-stimulatory domain (e.g., CD28) was added between the transmembrane and the CD3z domains and was shown to resolve low proliferation issues associated with the first generation CAR T cells (Fig. 1).^13–16^ In 2011, clinical trials of second generation CAR T cells in chronic lymphocytic leukemia (CLL) and B-cell acute lymphoblastic leukemia (B-ALL) patients achieved unprecedented results including complete remission.^1,2,17^ Since then, CAR T therapy has revolutionized the field of cell-based immunotherapy, especially for hematologic malignances. The third generation CAR, characterized by the incorporation of two co-stimulatory domains (e.g., CD28 and OX40) between the transmembrane and the CD3z domains, was also developed and shown to further augment CAR T cell performance (Fig. 1).^18,19^

However, most of CAR T therapy's successes were in hematologic cancers. When facing solid tumors, CAR T cells exhibited limited therapeutic efficacies, mostly attributed to difficulties in homing, infiltration, and survival in the immune-suppressive tumor microenvironment (TME), as well as tumor antigen escape and heterogeneity.^20–22^ Additionally, side effects associated with CAR T therapy have been reported, including on-target off-tumor toxicity, neurologic toxicity, cytokine release syndrome (CRS), etc.^23–25^ There is hence an urgent need for the development of the so-called next generation CAR T cells, with the engineering objectives to (a) enhance the efficacy of CAR T cells to overcome issues regarding the ineffectiveness of CAR T therapy in solid tumors, and (b) improve the safety of CAR T cells to mitigate and/or minimize the adverse toxicities associated with previous CAR T products.^26–29^ Tremendous efforts have been made toward these directions. Herein, we provide a review of recent novel strategies in CAR T cell designs that aim to improve the efficacy and safety of CAR T therapy. First, we discuss modifications on the CAR molecule at the ectodomain, transmembrane domain, and endodomain, as well as those involving multiple domains. We then review modifications on non-CAR molecules, either as “add-ons” to enhance CAR T cell performance, or as knock-out of negative regulators. In addition, we introduce inducible CAR T designs that allow spatial and temporal control over CAR expression or T cell activation. Finally, we discuss the applications of CAR in other types of immune cells. These innovations should aid the advancement of CAR T therapy particularly for treating solid tumors.

## MODIFICATIONS ON THE CAR MOLECULE

**FIG. 2.**
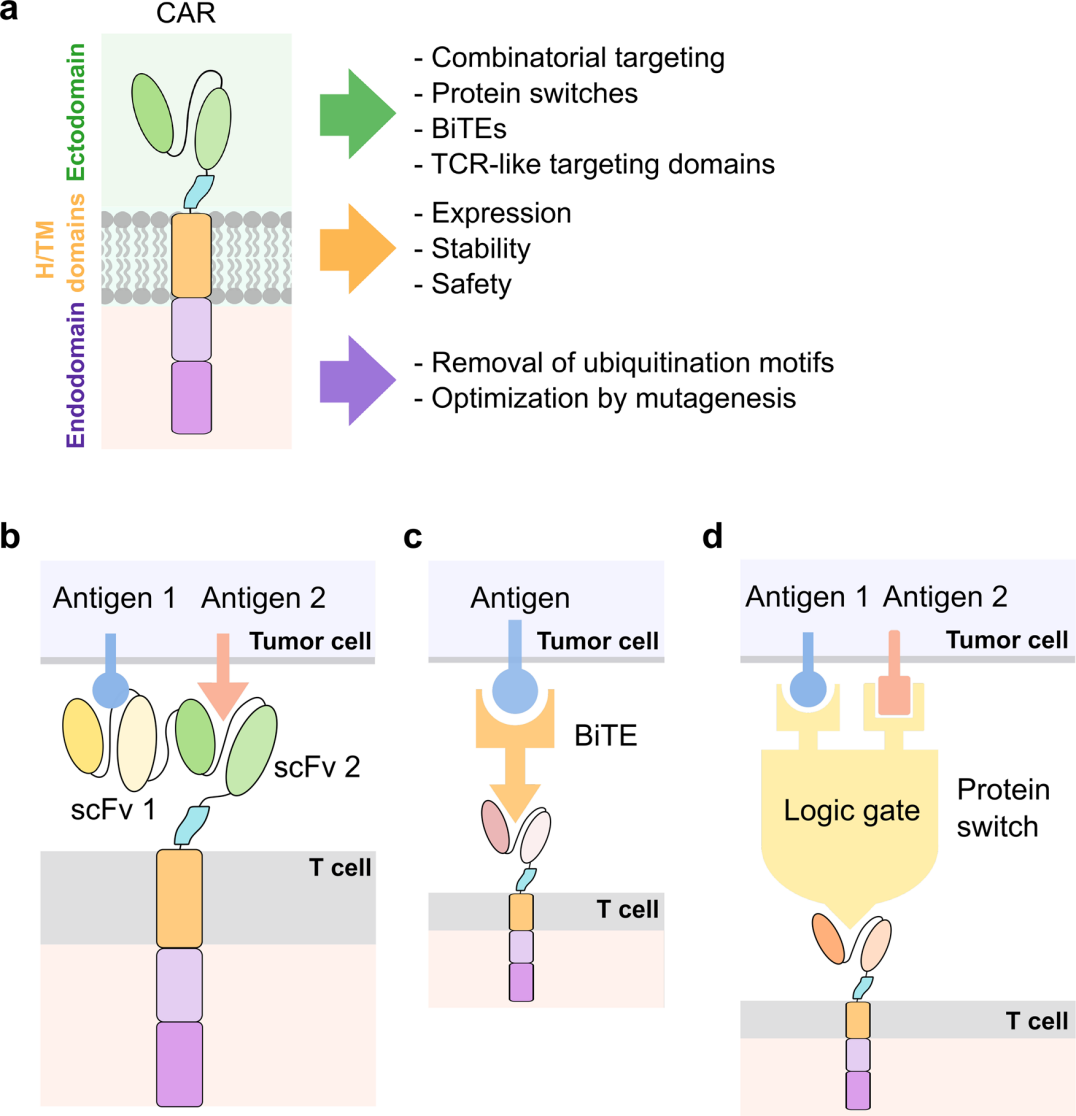
Engineering the CAR molecule. (a) Modifications on the ectodomain, hinge (H), and transmembrane (TM) domains, and endodomain of the CAR molecule. (b) Design of bispecific CARs. (c) Design of CARs utilizing bispecific T cell engagers (BiTEs) as adaptors between CAR T and tumor cells. (d) Design of CARs implementing protein switch-based logic gates to incorporate inputs from two or more tumor antigens.

The CAR molecule typically consists of the ectodomain, the hinge (H) and transmembrane (TM) domains, and the endodomain. The ectodomain is a key region responsible for the recognition of target antigens; the H/TM domains transmit antigen recognition signals to the endodomain where signaling occurs; and the endodomain is responsible for co-stimulatory signals that promote T cell survival and proliferation and stimulatory signals required for cytotoxic T cell responses. Multiple strategies of modifications and optimizations have been developed and applied to a constitutively expressed CAR. This section focuses on enhancements that occur in one or more domains in the CAR molecule [Fig. 2(a)].

### The ectodomain

Researchers have developed strategies to engineer antigen recognition to enhance CAR T targeting. For example, bispecific CARs containing two tandem ligand-binding domains have been shown to effectively implement an OR logic gate in CAR T signaling, where only two different types of antigens are capable of activating the CAR receptor [Fig. 2(b)]. A BCMA/CS1 bispecific CAR was shown to outperform T cells co-expressing separate BCMA and CS1 monospecific CARs.^30^ Thus, the enhanced avidity for the targeted cancer cells imparted by the bispecific CAR appears to enhance the immunotherapy and help avoid antigen escape that can occur with heterogeneous tumors. This strategy is generalizable to other combinations of antigens as shown by ongoing research in the area that reports comparable results.^31–33^ Some researchers have also explored universal CAR designs that utilize bispecific T cell engagers (BiTEs) as a bridge between CAR T and tumor cells [Fig. 2(c)]. For example, Kim *et al.* developed CAR T cells targeting fluorescein isothiocyanate (FITC), and used bispecific adapters consisting of FITC-conjugated folate to redirect the anti-FITC CAR T cells to tumor cells expressing folate receptors.^34^ Lee *et al.* further characterized FITC-folate mediated CAR T cells *in vivo* and demonstrated their ability in mitigating CRS.^35^ Rodgers *et al.* developed peptide-specific switchable CAR T cells (sCAR-T) recognizing peptide neo-epitopes (PNE) inserted in a tumor-antigen-specific antibody and demonstrated PNE dose-dependent activation of sCAR-T.^36^ Viaud *et al.* further optimized sCAR-T and characterized their antitumor ability in a syngeneic murine tumor model.^37^ Using similar principles, Paul *et al.* showed that a bispecific antibody targeting TRB5–5 and TRBV12 could specifically lyse malignant T cell lines in mouse models.^38^ Cho *et al.* developed a split, universal, and programmable (SUPRA) CAR system composed of a universal receptor (zipCAR) expressed on T cells, where a tumor-targeting scFv adaptor (zipFv) enabled the switch of targets in tumor cells and the response to multiple antigens using different adaptors without reengineering the T cells.^39^ These innovations provide solutions to antigen limitations in conventional CARs.

Despite the enhanced performance of bispecific CARs, for some applications, an OR-gate contributes to increased levels of on-target off-tumor toxicity leading to a lower therapeutic index. As it is difficult to identify surface antigens unique to cancer cells, CAR T cells are expected to kill normal cells expressing target antigens. For applications where such overlap in antigen expression occurs between cancerous and healthy tissues, more complex logic gates may be required. AND logic gates can require multiple antigens to be expressed on a cell before a T cell response can be triggered, and NOT logic gates can prevent CAR T activation when certain antigens are expressed on normal tissues/organs. Toward the implementation of more complex logic gates, Lajoie *et al.* developed a CAR that targets a non-native epitope that exists in a colocalization-dependent protein switch called Co-LOCKR [Fig. 2(d)].^40^ This switch can be toggled between a conformation where the epitope is exposed and another conformation where the epitope is hidden depending on co-localization of switch components. While this strategy has been shown to work *in vitro* to implement complex combinations of AND, OR, and NOT logic gates, such a strategy would require intravenous administration of the switch components.^40^ Thus, pharmacokinetics and immunogenicity of the colocalization-dependent protein switch may present future challenges for this approach. Other groups have also worked toward introducing Boolean logic gates in CAR T cells. For example, Salzer *et al.* have developed an avidity-based system where T cells are transduced with multiple CARs that bind weakly to targeted antigen. Thus, on their own, an individual CAR will be insufficient to mount a significant immune response. Then, by further engineering intermolecular interactions between such low-affinity CARs, the researchers were able to develop AND logic gates by introducing a dimerization domain.^41^

### The hinge and transmembrane domains

CARs can also benefit from optimizations in the H/TM domains. It has been shown that different H/TM domains can affect the expression and stability of the CAR molecules as well as the efficiency of signal transmitting.^42^ Some H/TM domains also appear associated with neurologic toxicity in CAR T therapies. Brudno *et al.* reported that the occurrence of neurologic toxicity is significantly lower in patients treated with Hu19-CD828Z CAR T cells than those treated with FMC63-28Z CAR T cells.^43^ As Hu19-CD828Z CAR differs from FMC63-28Z CAR in that it contains a human scFv as opposed to a mouse scFv, and H/TM domains from CD8α as opposed to from CD28, the authors hypothesized that the structural characteristics and the intramolecular and intermolecular binding of the H/TM domains caused the differences in cytokine release and neurologic toxicity between the two types of CAR T cells.^43^ Muller *et al.* also noted that the CD28 TM domain can promote heterodimerization with endogenous integrins.^44^ In another study, Ying *et al.* set off to optimize the CD19-BBz CAR used in the FDA-approved CAR T therapy medication CTL019 (Kymriah), as CTL019 was shown to cause toxic side effects in patients.^45^ By varying the length of the H/TM domains, they identified CD19-BBz(86) CAR as the top candidate and showed that CD19-BBz(86) CAR T cells were of similar potency but significantly reduced toxicities in a phase 1 trial.^45^ Thus, optimization of the H/TM domains can enhance the function and safety of CAR T cells.

### The endodomain

As the CAR endodomain is responsible for intracellular signaling, CAR function will be very dependent on the design of the endodomain. For example, Li *et al.* noticed that upon tumor engagement, expression of CARs in T cells would decrease over time. Specifically, they noticed that ubiquitination of the CAR led to endosomal recycling and loss of CAR expression.^46^ To counteract this observation, Li *et al.* mutated lysines in the endodomain to prevent ubiquitination. The end result was a CAR that outperformed the un-optimized CAR. Surprisingly, the ubiquitination-resistant CAR was able to achieve this with lower surface expression than the ubiquitination-susceptible CAR.^46^ Other researchers have taken a reductionist approach to remove components from the CAR and determine which domains are required for a T cell response. For example, Feucht *et al.* mutated immunoreceptor tyrosine-based activation motifs (ITAM) in CD3ζ to evaluate which ITAMs are essential for cytotoxicity. The results revealed that mutating the tyrosines to phenylalanines in ITAMs 2–3 resulted in strong effector responses and outperformed the standard 1928z CAR.^47^ This optimization enhanced the persistence of the CAR while retaining CAR function. Further still, Wu *et al.* have identified additional CD3 domains that may be useful for CAR T immunotherapy. Specifically, they showed that a CD3ε domain with its intracellular tail and the potential binding partners was capable of improving the cytotoxic activity of a second generation CAR.^48^

### Other modifications

Some researchers proposed to compartmentalize the co-stimulatory and activation signaling domains, which are commonly integrated into the same CAR molecule in second and third generation CAR designs. By separating CD3z signaling and CD28-mediated co-stimulatory signaling, Wilkie *et al.* developed dual antigen targeting CAR T cells whose proliferation required stimulation of both tumor antigens 1 and 2 (ErbB and MUC1).^49^ Kloss *et al.* engineered T cells expressing a CAR that only provides suboptimal activation signaling upon target antigen binding of prostate stem cell antigen (PSCA), and a chimeric co-stimulatory receptor that recognizes a different tumor antigen prostate-specific membrane antigen (PSMA). These engineered T cells only destroy prostate tumors expressing both PSCA and PSMA antigens but not single-antigen positive tumors.^50^ These combinatorial strategies that require the presence of dual or multiple tumor antigens to unleash the full potentials of engineered T cells can greatly reduce the on-target off-tumor side effects and broaden the applicability of tumor-associated antigen (TAA)-targeted T cell therapies.^51,52^

Some researchers have decided to forgo the typical CAR architecture altogether. For example, Liu *et al.* developed a chimeric receptor that utilizes an immunoglobulin heavy chain fused to TCR-Cα and an immunoglobulin light chain fused to TCR-Cβ.^53^ They showed that this double chain design mimicking the TCR architecture lacks the tonic signaling that poses challenges for some CAR designs. Additionally, the TCR-like scheme showed higher sensitivity toward targeted antigens than traditional CAR designs.^53^ Walseng *et al.* showed similar results in T cells and natural killer (NK) cells with their version of a TCR CAR that introduced a cysteine to enhance TCR dimer stability.^54^ Although promising in mouse models, safety concerns still exist for both TCR-based and CAR-based designs. In general, improving sensitivity toward a targeted antigen may result in a therapy with excessive on-target off-tumor toxicity due to overlap in antigen expression between healthy and cancerous tissues.

## MODIFICATIONS ON NON-CAR MOLECULES

**FIG. 3.**
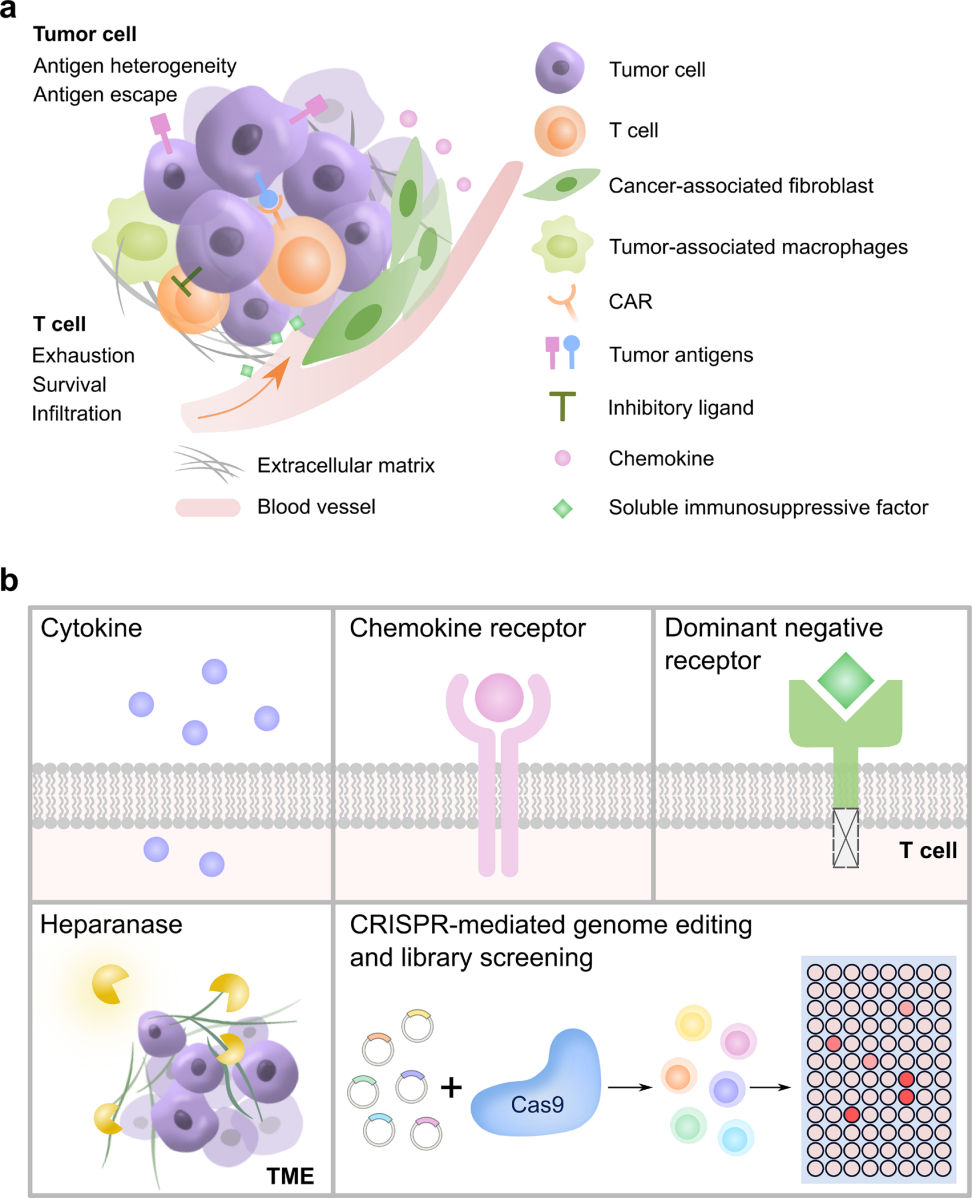
Engineering of armored CAR T cells through modifications on non-CAR molecules. (a) The immunosuppressive TME of a solid tumor faced by CAR T cells. (b) Representative approaches aimed to help CAR T cell fight the immunosuppressive TME, including expressions of cytokines, chemokine receptors, dominant-negative receptors, heparanases, and CRISPR-mediated genome editing and library screening in T cells.

Although CAR T cells have shown great success in the treatment of hematological malignancies, this approach has limited efficacy toward solid tumors. The immunosuppressive TME hinders the infiltration of the T cells, accelerates their exhaustion, and threatens their survival [Fig. 3(a)].^20^ For instance, the TME of solid tumors is enriched with extracellular matrix (ECM) proteins blocking the infiltration of T cells from blood vessels following intravenous administration. Another prominent example is the elevated expression of inhibitory ligands (e.g., PD-L1) on tumor cell surface that triggers inhibitory signaling pathways in T cells to compromise T cell function. Other challenges such as tumor antigen escape and antigen heterogeneity remain as well. Efforts have been attempted to engineer the so-called armored CAR T cells which can enhance the CAR T cell function in TME via the co-expression or knock-out of non-CAR molecules (see review in Ref. 39).^55^ Below we highlight some examples of these modifications [Fig. 3(b)].

### Cytokine secretion

Recombinant interleukin (IL)-12 has been used clinically to treat multiple types of solid tumors. Yeku *et al.* developed anti-ovarian tumor CAR T cells capable of constitutively secreting IL-12, and observed enhanced survival of these armored CAR T cells in the inhibitory TME of murine ovarian peritoneal carcinomatosis.^56^ It has also been reported that CAR T cells cultured in IL-15 can preserve a less-differentiated stem cell memory (Tscm) phenotype with reduced exhaustion and enhanced proliferation upon antigen engagement.^57^ In fact, multiple cell types, including macrophages and dendritic cells, can produce IL-15, which can stimulate CD8+ T cells and NK cells with increased proliferation and tumor cytotoxicity.^58^ Incorporating IL-15 production within GD2-targeting CAR T cells has demonstrated superior antitumor activity both *in vitro* and *in vivo* compared to conventional CAR T cells.^59^ Cytokines that are essential for T cell zone formation and maintenance in lymphoid organs can also be incorporated to enhance the therapeutic effects of CAR T cells. Adachi *et al.* engineered CAR T cells expressing both IL-7 and CCL19 (7 × 19 CAR T) and demonstrated complete regression of solid tumors in mice with prolonged survival as compared to conventional CAR T therapy.^60^ More recently, Luo *et al.* further engineered CAR T cells expressing IL-7 and CCL21 (7 × 21 CAR T), which revealed superior therapeutic efficacy against solid tumors to conventional CAR or 7 × 19 CAR.^61^

### Chemokine receptor

Insufficient tumor-directed trafficking is another key factor that limits cell-based immunotherapy against solid tumors. To overcome this, scientists have engineered CAR T cells that can utilize the tumor-secreted chemokines for their homing to the tumor sites. For instance, Hodgkin lymphoma can produce chemokine CCL17 and CCL22 which are attractants for the CCR4-expressing T helper 2 (Th2) and regulatory T (Treg) cells, but not for the CD8+ T cells, which lack CCR4 expression. Based on that, researchers have engineered CCR4-expressing CAR T cells, which have shown improved homing and antitumor activity when infused intravenously in mice engrafted with human Hodgkin lymphoma.^62^ Similarly, IL-8 has also been reported as a chemoattractant for neutrophils and myeloid-derived suppressive cells which contribute to the immunosuppressive nature of TME. Co-expression of IL-8 receptor CXCR2 in the CAR T cells has also achieved promising outcomes including enhanced tumor homing and efficacy in multiple solid tumor models.^63–65^ Besides, co-expression of other chemokine receptors, including CCR2b,^66^ CXCR3,^67^ CXCR4,^68^ and CCR6,^69^ have also shown promising results in solid tumor treatment.

### Heparanase

Endogenous T cells can produce enzymes that degrade the ECM of solid tumors, one of the barriers for T cell infiltration; however, it has been found that CAR T cells can lose such capability of degrading ECM during the *ex vivo* engineering process. To improve the tumor infiltration of CAR T cells, Caruana *et al.* reequiped the anti-GD2 CAR T cells with the enzyme heparanase (HPSE), which degrades heparan sulfate proteoglycans, the main components of ECM.^70^ Based on their results, these CAR T cells indeed demonstrated improved infiltration into xenograft tumors in mice and prolonged survival as compared to CAR T cells lacking heparanase expression.

### Dominant-negative receptors

Another approach to overcome the immunosuppressive signals within TME is to block the immunosuppressive pathways in CAR T cells. Transforming growth factor β (TGF-β), secreted by many tumors including prostate cancer, is known to potently suppress the immune system, creating an immunosuppressive milieu within solid tumors. Studies have demonstrated that the TGF-β signaling can be blocked by expressing dominant-negative TGFBRII, which lacks the intracellular domain for downstream signaling. Using this as an add-on, Kloss *et al.* demonstrated that the potency of PSMA-directed CAR T cells can be greatly enhanced, with increased T cell proliferation, cytokine secretion, resistance to exhaustion, and long-term *in vivo* persistence.^71^ Similarly, co-expressing PD-1 dominant-negative receptor, which blocks the PD-1 pathway, has also demonstrated augmented efficacy for various CAR T therapies targeting CD19, mesothelin, and HIV-1.^72–74^ Compared with antibody-based PD-1 blockade, the genetic-engineering approach can provide more sustainable and tumor-limited effects and has provided opportunities to treat different types of solid tumors.

### High-throughput pooled knock-in

The add-on expression of endogenous or exogenous genes has shown great promise for cancer immunotherapy, and in fact decades of studies on T cell signaling and function have suggested numerous candidates for such applications. However, a high-throughput method for screening the gene candidates that most potently enhance the performance of cell therapies is still needed. Recently, Roth *et al.* developed a high-throughput platform to assess the functional effects of pooled library of knock-in gene templates in the same locus through CRISPR targeting, with which they demonstrated the rapid screening of a barcoded 36-member library that included dominant-negative receptors, synthetic switch receptors, transcription factors, and metabolic regulators/receptors.^75^ Using a human melanoma mouse model allowed the direct comparison of T cells knocked in with the pooled library and identified subsets of knock-in constructs that promoted *in vivo* tumor infiltration. Particularly, the TGF-βR2–41BB chimeric receptor, one of the candidates in the library, has been shown to enhance T cell fitness and promote expression of key effector cytokines, and improve solid tumor clearance *in vivo*, suggesting that the pooled knock-in technology can be a powerful tool in identifying potential lead constructs from large libraries.

### Knock-out of inhibitory surface receptors

Alternatively, researchers have investigated the deletion or disruption of genes that negatively regulate T cell performance. Immune checkpoints such as PD-1 and CTLA-4 are inhibitory receptors that can suppress T cell activation and promote T cell exhaustion and dysfunction.^76,77^ Immune checkpoint blockade therapies utilizing monoclonal antibodies against the checkpoint receptors have shown promising clinical results.^76,77^ Investigators have also applied immune checkpoint blockade in CAR T therapy by combining CAR T treatment with PD-1 blocking antibody administration,^78^ rewiring PD-1 or CTLA-4-based inhibitory signals to CAR T activation (iCARs),^79^ or engineering CAR T cells with constitutive anti-PD-1 scFv expression and secretion.^80^ Moreover, recent advancement in gene editing technology has allowed the manipulation of endogenous genes. Using the CRISPR/Cas9 system, Su *et al.* performed PD-1 gene knock-out in patient-derived T cells and observed enhanced cytokine production and cytotoxicity *in vitro.*^81^ Rupp *et al.* further applied CRISPR/Cas9-mediated PD-1 knock-out in anti-CD19 CAR T cells and demonstrated improved clearance of PD-L1+ tumor xenografts *in vivo.*^82^ Hu *et al.* observed similar results with PD-1 knock-out in anti-mesothelin CAR T cells.^83^ Furthermore, Ren *et al.* performed multiplex genome editing to simultaneously knock-out TCR, HLA class I molecule, and PD-1, generating universal allogeneic PD-1 deficient CAR T cells with enhanced antitumor activity.^84^ Disruption of other inhibitory receptors such as CTLA-4 has also been studied in CAR T cells and achieved promising results.^85,86^ A list of potential immunoinhibitory receptor targets for genome editing in CAR T cells has been reviewed elsewhere.^87^

### Knock-out of negative regulators

Meanwhile, systematic screening methods have been developed to identify key negative regulators in T cells. Shifrut *et al.* performed CRISPR/Cas9-based genome-wide loss-of-function screens and identified negative regulators of proliferation following stimulation in primary human T cells *in vitro.*^88^ Wei *et al.* developed a pooled CRISPR/Cas9 mutagenesis screening approach and identified REGNASE-1 as a major negative regulator of T cell antitumor activity among 3017 metabolism-associated factors.^89^ REGASE-1 knock-out T cells demonstrated improved accumulation and persistence in an adoptive cell therapy model *in vivo.*^89^ In another case report, Fraietta *et al.* discovered unintended disruption of the methylcytosine dioxygenase *TET2* gene by CAR lentiviral integration in a CAR T cell administrated into a chronic lymphocytic leukemia (CLL) patient, in addition to a hypomorphic variant in the other *TET2* allele.^90^ The TET2-disrupted CAR T cell underwent massive *in vivo* expansion and became the dominant population (94% of the CD8+ CAR T cell repertoire at the peak of response), leading to complete remission in this patient.^90^ Further analysis revealed epigenetic reprogramming and central memory phenotype of the TET2-disrupted CAR T cells, whose potency-enhancing effect was recapitulated by experimental knockdown of TET2.^90^ While TET2 is a tumor suppressor gene and extreme caution should be used when disrupting such genes, these reports highlighted the potential of endogenous gene silencing and genome/epigenome editing in enhancing the efficacy of CAR T therapy.

### Off-the-shelf CAR T cells

An additional hurdle that CAR T based therapies must overcome involves the manufacture and delivery of autologous CAR T cells. FDA-approved therapies require taking a patient's own cells, reprogramming them, and allowing them to proliferate prior to injecting the modified cells back into the patients. The whole process may need around two weeks. For many patients who failed prior to front-line therapies, they may not survive long enough to receive and benefit from the reprogrammed cells. Thus, another field of research involves developing off-the-shelf allogeneic CAR T cells that do not require a two-week waiting period.

Allogenic donor CAR T cells may attack recipient tissues and cause graft-vs-host disease (GVHD) mediated by the TCR expression on donor T cells.^91^ One solution is to knock-out TCR in allogenic donor CAR T cells. Poirot *et al.* utilized transcription activator-like effector nuclease (TALEN)-mediated gene editing approach to knockdown TCR and CD52 (a protein targeted by chemotherapeutic agent) in CD19CAR T cells, and demonstrated their high efficacy in a blood cancer mouse model.^92^ This universal CD19CAR T product (later termed UCART19) was later used to treat B-ALL in two infants,^93^ followed up by two phase 1 studies in pediatric and adult patients.^94^ Other attempts to generate universal CAR T cells include CRISPR/Cas9-mediated triple knock-out of TCR, HLA, and PD1,^84^ as well as the integration of CAR into the TCR alpha constant (TRAC) locus.^95,96^ In the event that GVHD or other serious adverse events occur that require termination of the CAR T therapy, researchers are actively developing safety switches to kill or inactivate the allogeneic CAR T cells.^97–99^

At the same time, host-vs-graft (HVG) activities triggered by the infusion of allogeneic T cells, including the host T- and NK-cell responses to eliminate foreign cells, may limit the persistence of infused CAR T cells, and should also be minimized to achieve full therapeutic benefits. Ayuk *et al.* reported that for some patients, allogeneic CAR T therapy may be feasible as CAR T cells from an HLA-matched donor were able to proliferate and persist during immunotherapy.^100^ In addition, the allogeneic T cell-activated host lymphocytes would upregulate surface receptors, such as 4–1BB (CD137), which can serve as markers to distinguish the activated cytotoxic effectors from the unstimulated populations. Based on that, Mo *et al.* engineered the chimeric 4–1BB-specific alloimmune defense receptor (ADR) which selectively eliminates the activated host lymphocytes by the allogeneic CAR T cells while sparing the resting lymphocytes.^101^ They showed that the ADR-expressing CAR T cells can successfully evade immune rejection and achieve sustained tumor eradication in mouse models of allogeneic T cell therapy of hematopoietic (CD19 CAR) and solid (GD2 CAR) cancers.

## STIMULI-INDUCIBLE CAR T CELLS

**FIG. 4.**
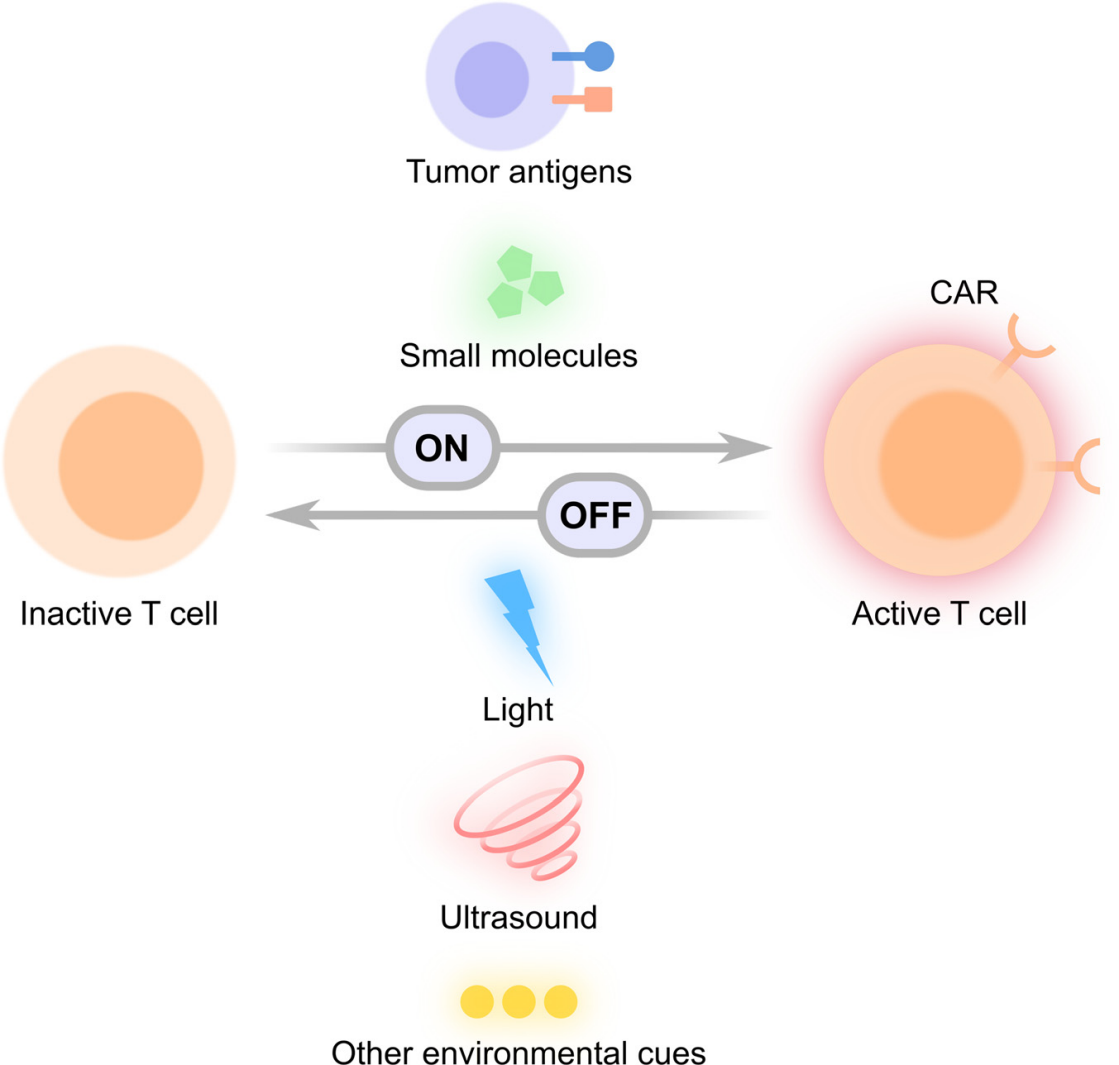
Conceptual illustration of stimuli-inducible CAR T cells. Various ON- and OFF-switches controlled by external stimuli such as tumor antigens, small molecules, light, ultrasound, and other environmental cues have been utilized to activate functional CAR expression (ON-switch) or deactivate CAR-expressing T cells (OFF-switch).

Toxicity remains a major concern in CAR T therapy. CRS is a potentially life-threatening side effect characterized by systemic elevations of cytokines such as IL-6 and interferon γ (IFN γ). CRS is one of the most common adverse effects in CAR T therapy caused by infusion of large amounts of active CAR T cells and is commonly treated with IL-6 receptor blockade.^102–104^ Another potentially lethal side effect is the “on-target off-tumor” toxicity, where CAR T cells attack normal tissues expressing low levels of the target antigen. An infusion of 10^10^ anti-ERBB2 CAR T cells attempted to treat a patient with metastatic ERBB2+ cancer caused acute respiratory distress and the subsequent death of the patient.^105^ The serious toxicity was later believed to be caused by the recognition of low levels of ERBB2 on lung epithelial cells by the infused CAR T cells.^105^ There is hence an urgent need to engineer safer CAR T cells whose activation can be tightly controlled spatiotemporally to prevent CRS, on-target off-tumor toxicity, and other adverse effects. To this end, researchers have developed stimuli-inducible CAR T cells, where functional CAR expression is activated or deactivated in a controllable manner by environmental cues such as tumor antigens, small molecules, light, ultrasound, and other stimulations (Fig. 4). These designs provide enhanced controllability of CAR T cells, bringing the field one step closer to safer CAR T therapy.

### Automated CAR T cells

**FIG. 5.**
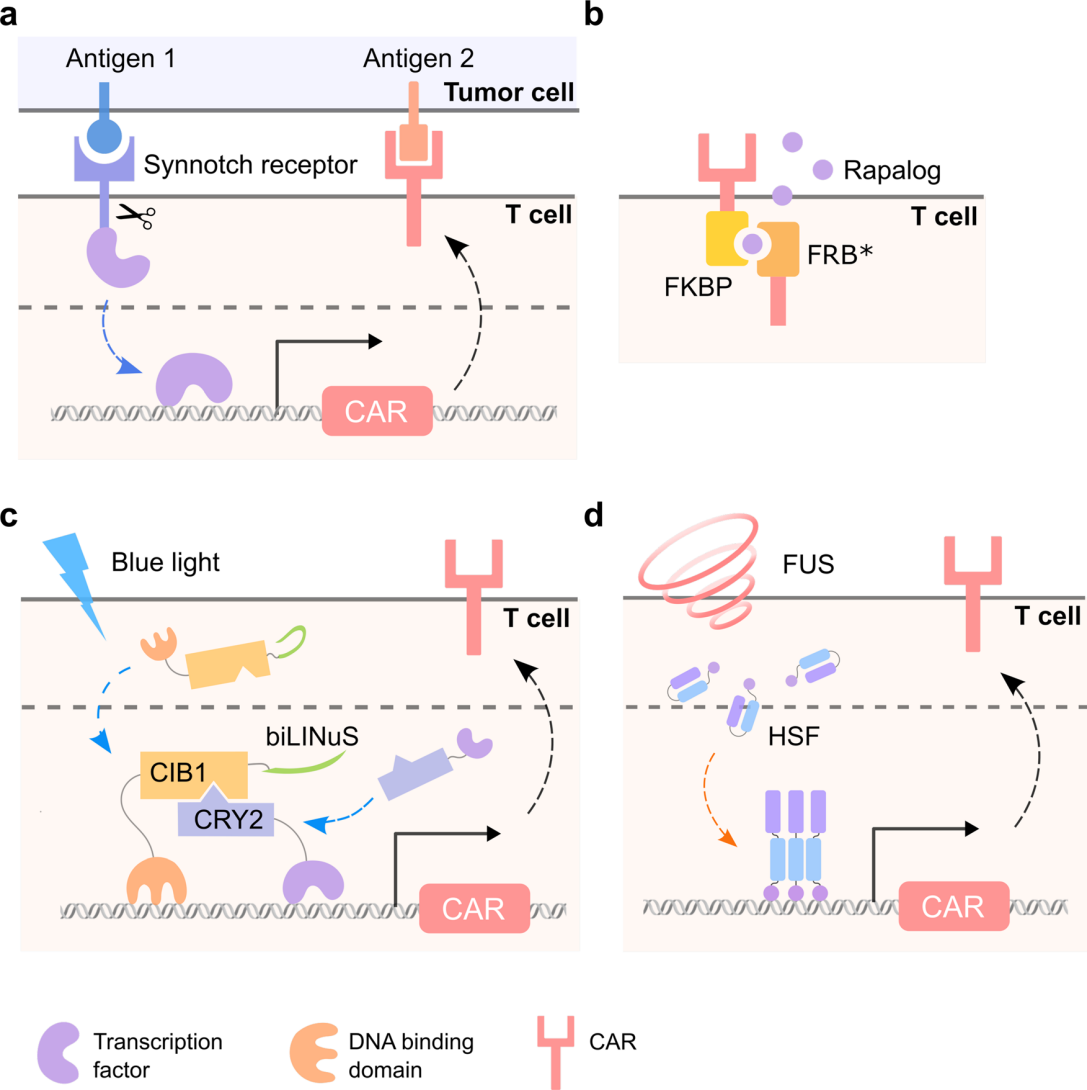
Representative designs of stimuli-inducible CAR T cells. (a) Synnotch CAR T cells. (b) Rapalog-inducible CAR T cells. (c) Blue-light-controllable CAR T cells with the LINTAD system. (d) FUS-controllable heat-sensitive CAR T cells.

The engineering of AND-gate T cells based on the synthetic Notch receptor (synNotch) system which is activated only by dual antigen recognition allows precise control of CAR T cell signaling [Fig. 5(a)]. In the synNotch design, the CAR-like synNotch receptor on the T cells senses antigen 1 presented on the tumor cells, which induces the expression of a CAR targeting tumor antigen 2; thus, only the tumor cells expressing dual antigens are eliminated.^106^ This AND-gate circuit expands the antigen sets on solid tumors that can be targeted safely with CAR T cells.^107^ For instance, the synNotch CAR T cells can sense ROR1 protein and induce the expression of CAR molecules specific for EpCAM or B7-H3, which are expressed on ROR1+ tumor cells but not ROR1+ stromal cells; therefore, this system can overcome the lethal toxicity of constitutive ROR1-CAR T cells.^108^ Furthermore, the synNotch CAR T cells that recognize the combination of alkaline phosphatase placental-like 2 (ALPPL2) and the tumor-associated antigen-melanoma cell adhesion molecule (MCAM), mesothelin, or HER2 were demonstrated to more precisely guide the T cells to target the tumors.^109^ Recently, T cells with multiple synNotch receptors as flexible regulatory connectors were developed, which could achieve precise tumor recognition by integrating up to three different antigens while ignoring related two-antigen tumors.^110^ Similar approaches also allowed the integration of signals from the recognition of multiple imperfect but complementary antigens for the design of T cell killing functions.^111^ Mechanistically, in addition to the precise targeting of specific tumor cells, the synNotch-regulated CAR expression has also been shown to avert tonic signaling and exhaustion, maintain a higher fraction of the T cells in a naïve/stem cell memory state, thus leading to more efficient tumor killing than conventional CAR T cell.^109,111^

Another approach of automated conditionally expressed CARs is driven by tumor microenvironment-associated factors such as hypoxia. Using quantitatively characterized hypoxia-responsive element (HRE) together with a panel of core promoters, Ede *et al.* found the synthetic promoter, YB_TATA, to have the most prominent contrast in inducing reporter gene expression between normal and hypoxia conditions.^112^ These regulatory elements were further applied to engineer hypoxia-inducible CAR T cells that can be conditionally activated in the hypoxia tumor microenvironment.^112^ Later, instead of inducible expression of CARs directly by the hypoxia-responsive element, the oxygen-dependent degradation domain (ODD) of HIF1a was also utilized to induce the degradation of the fused CARs under normoxia condition and thus achieving T cell activation and tumor killing only under hypoxia conditions.^113^ Recently, a dual oxygen-sensing CAR T cell was developed by appending the ODD domain onto the CAR, whose expression is driven by nine consecutive HREs.^114^ This design allows the inducible expression of CAR under hypoxia and the degradation of CAR expression leakage under normoxia environment.

### Small molecule-controllable CAR T cells

Chemical controllable CAR is an alternative way to control the dose and timing of activated T cells to mitigate side effects such as CRS. A robust ON-switch split-CD19CAR design based on FKBP-FRB* dimerization was proposed, in which the functional CAR molecules are formed only after the addition of dimerization small molecule rapamycin analog AP21967 (rapalog), producing titratable, reversible, and temporally controllable CAR T cells^115^ [Fig. 5(b)]. This study provides an elegant example of designing safer therapeutic cells by integrating autonomous and user controls. Similar methods can be extended to a broader range of small molecule-inducible dimerization systems^116,117^ or other CAR molecules such as the epidermal growth factor receptor variant III (EGFRvIII) CAR.^118^ Another ON-switch design utilizing the tetracycline (Tet)-On system, an inducible gene expression system for mammalian cells, was also used to control CAR expression with a small molecule doxycycline (Dox) and achieved low background expression and an equivalent killing efficiency to the conventional CAR in the presence of Dox.^119^ Yang *et al.* designed an inducible gene expression system based on a dietary molecule resveratrol (RES) to regulate CAR expression and demonstrated that RES-activated CAR T cells achieved *in vivo* cytotoxicity comparable to that of conventional CAR T cells.^120^

In addition to the ON-switches, the inducible suicide switch (OFF) has been applied to control CAR T cells,^121–126^ especially when these cells have adverse effects on human patients. One of the most successful suicide genes is the inducible caspase-9 (iCasp9) gene, where the dimerization of iCasp9 by a small molecule compound can trigger the apoptosis of 90% of the modified T cells within 30 min in GVHD patients and end GVHD without recurrence.^124^ However, the suicide switch approach is irreversible, eliminating the entire CAR-positive T cell population. Yang *et al.* showed that RES-based inducible gene expression system can also be used to reversibly repress CAR expression, serving as an OFF switch.^120^ An alternative approach is an inducible degradation of CARs in T cells using the ligand-induced degradation (LID) domain such as degron^127,128^ and dihydrofolate reductase (DHFR) destabilizing domain.^128^ The addition of a small molecule ligand of degron-shield-1 promotes the proteasomal degradation of the CAR-LID fusion protein.^127^ In contrast, CAR-DHFR can be stabilized by the FDA-approved antibiotic trimethoprim (TMP),^128^ achieving the drug-dependent control of CAR expression and activity both *in vitro* and *in vivo*. Jan *et al.* also developed an OFF-switch CAR using the clinically approved drug lenalidomide to trigger the degradation of CAR proteins.^117^ Additionally, the proteolysis-targeting chimera (PROTAC) compound was also applied to control the CAR molecules.^129^ PROTACs are small bifunctional molecules that are able to bind to target protein ligands and E3 ubiquitin ligase, which resultantly degrades the target protein through the ubiquitin-proteasome system.^130^ The CD19 CAR molecule linked with the bromodomain (BD) from BRD4 protein can be degraded by the E3 ligases after the addition of PROTACs; however, the problem of PROTACs is the degradation of endogenous proteins as well as CAR protein, which may be toxic to CAR T cells.^130^

In addition to their usage in inducible CARs, small molecules were also applied to pre-condition the CAR T cells before transplantation to enhance therapeutic efficacy. Dasatinib, an FDA-approved tyrosine kinase inhibitor that was initially used to treat chronic myeloid leukemia (CML), was found to be effective in temporarily inactivating CAR T cells. The treatment of dasatinib reduced the acute toxicity, inhibited the tonic CAR signaling, and reinvigorated exhausted CAR T cells, thus resulting in superior antitumor responses *in vivo*.^128,131,132^ Similarly, after low dose treatment of Decitabine, a DNA methyltransferase inhibitor that is FDA-approved for treating myelodysplastic syndromes (MDS), CAR T cells showed higher expressions of memory-, proliferation-, and cytokine production-associated genes, and enhanced their persistent antitumor capacity *in vivo*^133^ through epigenome reprogramming. Since these small molecules are FDA-approved and have demonstrated safety in humans, implementing these drugs as an on/off control in CAR T cell immunotherapy should be straightforward.

### Light-controllable CAR T cells

Optogenetics, where optical and genetic methods are combined to control biological processes with high spatiotemporal precision, was mainly applied in neurobiology in the early stage.^134^ With the development of genetically encoded light-sensitive proteins, optogenetics has become an increasingly popular tool for the remote control of cellular functions.^135^ Kennedy *et al.* demonstrated the application of blue-light-controllable dimerizers cryptochrome 2 (CRY2) and CIB1 in protein translocation, transcription, and Cre-mediated DNA recombination, opening doors for the remote control of cellular functions using a new generation of optogenetic tools.^135,136^ Recently, researchers have explored the application of optogenetics in CAR T therapy as it may offer enhanced safety and controllability. Huang *et al.* developed a light-inducible nuclear translocation and dimerization (LINTAD) gene activation system by integrating the CRY2-CIB1 dimerizer and the LOV2-based light-inducible nuclear localization signal (biLINuS) [Fig. 5(c)]. The LINTAD system was applied to regulate CAR expression in T cells and achieved blue-light-controllable tumor killing *in vivo.*^137^ Zhang *et al.* engineered a photoswitchable CAR based on the FITC-folate mediated CAR (reviewed above), where they inserted a photocleavable linker between FITC and folate moieties in the bispecific adaptor, allowing the deactivating of CAR T cells by light.^138^ O'Donoghue *et al.* engineered an optoCAR system by fusing one part of a split CAR to the improved light-inducible dimer (iLID) and another to the iLID binding partner SspB, allowing the reconstitution of a functional intact CAR upon illumination.^139^ Similarly, He *et al.* also engineered optoCAR based on an optical dimerization system with a circularly permuted LOV2 (cpLOV2), and demonstrated the photoinducible antitumor activity of the optoCAR T cells *in vivo.*^140^ While light offers precise spatial and temporal control, its limited tissue penetration depth may prevent further applications in human patients. To overcome this, technologies involving upconversion nanoparticles, implantable LEDs, near-infrared (NIR) light, and optical fibers have been developed.^141–143^ For example, Nguyen *et al.* utilized upconversion nanoplates and demonstrated antitumor responses of light-switchable CAR (LiCAR) T cells activated by NIR light.^144^

### Ultrasound and/or heat-controllable CAR T cells

Ultrasound can penetrate deep into biological tissues. In addition to its traditional usage as an imaging tool for diagnosis, ultrasound has been applied to regulate cellular functions for therapeutic purposes. Pan *et al.* utilized ultrasound to mechanically perturb microbubble-coupled cells, activating the mechanosensitive ion channel Piezo1 and subsequent molecular events including calcium influx, nuclear factor of activated T cells (NFAT) translation, and NFAT-mediated gene expression. They applied this system in CAR T cells and showed ultrasound-inducible tumor cell killing *in vitro.*^145^

In addition to direct mechanical stimulation, ultrasound can also cause local hyperthermia when the deposited mechanical energy is converted to thermal energy via internal friction. Focused ultrasound (FUS), capable of causing temperature elevation in a confined region, has been widely used to ablate tumors in the clinics. Inspired by the endogenous heat shock response where heat (or other stresses) activates the heat shock promoter (Hsp) through heat shock factors (HSFs) to drive the expression of heat shock proteins, researchers have employed FUS to activate Hsp-driven transgene expressions by generating mild hyperthermia *in vitro* and *in vivo.*^146–149^ Recently, Wu *et al.* developed FUS-CAR T cells containing an Hsp-driven Cre-lox switch that can be controlled by FUS to activate CAR expression.^150^ They further engineered a reversible FUS-CAR T cell where CAR production is directly driven by the Hsp [Fig. 5(d)]. The FUS-activated FUS-CAR T cells demonstrated antitumor efficacies in two subcutaneous tumor models; more importantly, the FUS-CAR T cells were shown to cause significantly lower on-target off-tumor toxicity compared with standard constitutive CAR T cells.^150^ In another Hsp-based design, Miller *et al.* utilized plasmic gold nanorods to convert near infrared (NIR) light to heat, activating Hsp-driven IL15 superagonist in constitutive CAR T cells. Their results revealed that NIR-activated IL15-expression enhanced the antitumor activity of CAR T cells *in vivo.*^151^

## CELL THERAPY BASED ON MACROPHAGES AND NATURAL KILLER CELLS

CAR T therapy remains less effective for solid tumors, mainly attributed to the antigen heterogeneity as well as the physical barrier and immune-suppressive tumor microenvironment.^152^ Solid tumor microenvironment (SME) can attract myeloid cells including macrophages through chemokines secreted by tumor or stroma cells.^153^ As such, there is abundant number of macrophages in SME. Typically, these macrophages are polarized toward M2 phenotype, which promotes tumors and resist therapeutic treatments by providing physical barrier and suppressive microenvironment.^154^ Approaches have been designed to deplete these suppressive M2 tumor macrophages to enhance the therapy efficacy.^155^ Recently, reengineering these tumor macrophages with CAR (CAR-M) has rendered impressive therapeutic efficacy against solid tumors.^156^ CAR-M cells had a tendency polarizing toward M1 antitumor phenotypes and also indirectly recruited host immunity to eradicate target tumors including ovarian cancer.^157^ In an alternative approach, *i*ntegrated sensing and activating protein (*i*SNAP), integrating the functionality of both protein-based biosensors and activators, was designed to rewire the negative “don't eat me” CD47/SIRPα pathway into activating and pro-phagocytic signaling in macrophages. The results showed that the *i*SNAP-rewired macrophages possessed a strong capability of negating the inhibitory CD47 signaling and eradicating tumors including non-Hodgkin's lymphoma (NHL) and colon cancer.^158^ Given the abundance of macrophages at SME, it becomes an attractive topic to convert the phenotypes and functions of these macrophages via genetic engineering for immunotherapy against solid tumors.

Nature killer (NK) cells are lymphocytes playing key roles in the innate immunity. About 10% mononuclear cells are NK cells in peripheral blood samples.^159^ NK cells are in general, insensitive to antigens presented by major histocompatibility (MHC) molecules. As such, NK cells can be engineered to develop allogenic therapeutic cell products, avoiding GVHD.^160^ Initial tests showed that CAR-NK rarely causes CRS. This is particularly appealing for the development of “off-the-shelf” therapeutic products.^161^ In fact, CAR-NK cells have been developed as potent tools against tumors.^162–165^ NK cells derived from human iPSCs were engineered to express CAR and demonstrate strong antitumor activity against ovarian cancers.^166^ Further engineering of these iPSC-derived NK-CAR cells by knocking out the gene encoding cytokine-inducible SH2-containing protein which regulates the IL-15 and JAK-STAT pathways, significantly enhanced the metabolic fitness of NK-CAR cells for more efficient toxicity against multiple cancer types.^167^ However, compared to T cells, NK cells are relatively more difficult to engineer genetically and to maintain expansion and persistence.^159^ More research activities will hence be warranted to further improve NK-CAR functionality.

In summary, CAR-M is advantageous over CAR T in terms of SME infiltration, but lacks the ability to proliferate, making cell number a limiting factor in CAR-M based therapies. CAR-NK has the advantages of causing less toxicities and significantly reduced risk of GVHD, rendering it a safer and more universal therapy. Its antigen-independent killing capability may also be harnessed to tackle the issue of antigen escape in some tumors. However, CAR-NK also suffers from difficulties in gene delivery and cell expansion. More optimizations are needed to make CAR-M and CAR-NK alternative and complementary treatments to CAR T therapy.

## CONCLUSIONS AND PERSPECTIVES

The fast development of CAR T therapy in the past decades is mainly driven by the interrelated feedback loops between clinical trials and laboratory research. Researchers gain new insights into CAR efficacy, toxicity, and resistance from the “bedside” and create new designs of CAR at the “bench” and then reapply these new CARs to the bedside. Multiple cutting-edge frontiers have shifted CAR designs from simply changing co-stimulatory domains to more sophisticated strategies.

The difficulties of CAR T therapy mainly lie in the treatment of solid tumors, especially in the balance between the reduction of on-target off-tumor toxicity and the persistence of T cells in the immunosuppressive tumor microenvironment.^152^ Modifications of players in key signaling pathways in CAR T cells have shown promising results, including increased trafficking and fitness of T cells.^89,168^ Meanwhile, controllable designs have demonstrated spatiotemporal precisions in the efficient regulation of T cells at the tumor site.^137,150,151^ CAR designs applied to other cell types such as macrophages^157^ and NK cells^166^ to circumvent the limitations of T cells are also leading to promising results.

However, most of the engineering strategies only focus on improving individual features. Presumably, combinations of multiple designs can equip the T cells with multiple layers of capabilities and may achieve better therapy results. For example, a combination of knock-out or knock-in with controllable T cells will increase T cell homing and fitness and confine T cell activity at the tumor site, thus reducing the off-tumor toxicities. In the future, high-throughput CRISPR-based screening in human T cells^88,169^ tailored for the controllable system may shift the CAR engineering direction and outcomes. Furthermore, due to the complicated biological crosstalk among different immune cells in the body, the combinations of different cell types for tumor treatment may lead to novel approaches with high efficiency and specificity. For instance, mixing engineered T cells with NK cells has shown promising antitumor efficacy in a multiple myeloma model.^170^ As new technologies and engineering approaches are rapidly evolving, it is envisioned that cell-based immunotherapy integrating engineering tools and synthetic biology will provide safe, efficient, and precise therapeutic options for a broad range of solid tumors.

## Data Availability

Data sharing is not applicable to this article as no new data were created or analyzed in this study.
